# Effect of an Effervescent Multivitamin on Color and Surface Roughness of Micro-Hybrid Dental Resin Composites

**DOI:** 10.3390/ma17051040

**Published:** 2024-02-24

**Authors:** Bengü Doğu Kaya, Pınar Yılmaz Atalı, Seda Özmen, Selinsu Öztürk, Bilge Tarçın

**Affiliations:** 1 Department of Restorative Dentistry, Faculty of Dentistry, Marmara University, Istanbul 34854, Turkey; 2 Department of Restorative Dentistry, Faculty of Dentistry, Yeditepe University, Istanbul 34728, Turkey; 3 Department of Restorative Dentistry, Institute of Health Sciences, Marmara University, Istanbul 34854, Turkey

**Keywords:** discoloration, effervescent multivitamin tablet, micro-hybrid composite, surface roughness, vitamin C

## Abstract

The use of multivitamins has become widespread globally, yet there is a scarcity of studies investigating their impact on resin composite restorations. This study aimed to evaluate the effect of an effervescent multivitamin tablet on micro-hybrid dental resin composites’ surface roughness and color. Fifty disc-shaped samples (8 × 2 mm, shade A2; *n* = 10) were prepared and polished using five different micro-hybrid resin composites (Pergamon, Dentac, Turkey; Estelite Posterior, Tokuyama, Japan; Geanial Anterior, GC, Japan; Charisma Opal, Kulzer, Germany; Beautifil II, Shofu, USA). Samples were immersed in 200 mL water to one effervescent multivitamin tablet (Redoxon Triple Action, Bayer) at 24 °C for 2 min a day in 24 h intervals for 30 days. All samples’ surface roughness (Ra) and ΔE(L*a*b) measurements were recorded at the beginning and end of the 30 days. The Wald chi-square and a two-way ANOVA were used for statistical analysis (significance level *p* < 0.05). The resin composite type and exposure to the multivitamin had a statistically significant effect on Ra values (*p* < 0.05). The resin composite type had a statistically significant effect on ΔE values, likely due to the higher mean value of BII (*p* = 0.040). The surface roughness and color of resin composites can be affected by multivitamins with a pH value of 3.0. Therefore, it is important to consider the patient’s routine vitamin intake during resin composite selection. Additional research is required to explore the properties of different dental restorative materials.

## 1. Introduction

The importance of nutrition for immune functions is well known in the world. It is believed that the use of certain vitamins, such as vitamin C, vitamin D, and zinc, may help reduce the chance of viral infection, so the population’s interest in taking multivitamins has increased [1]. Vitamin supplements can be found in various forms, such as liquids (syrup), pills, chewable tablets, gummies, lozenges, and effervescent tablets. Effervescent tablets are easy to take and contain organic acids (e.g., citric, tartaric, and maleic acids) and carbonate salts (sodium bicarbonate/carbonate or potassium bicarbonate/carbonate). These compounds form carbon dioxide when they come into contact with water [2]. Researchers have focused mostly on the effect of effervescent vitamins/multivitamins on dental hard tissue and have found that these tablets have an erosive potential due to their acidic content and resulting low pH [2,3]. However, their impact on dental resin composite restorations has not been adequately explored. Furthermore, dental resin composite materials are usually the favored option for restorations, and numerous studies have investigated these materials’ properties [4,5]. Although restorative materials are less susceptible to erosion than enamel, their clinical performance is also affected by erosion. It has been reported that acid has detrimental consequences for restorative materials’ physical and chemical properties [6], surface roughness, and microhardness [7]. In addition, these vitamin supplements can be an extrinsic source and may be responsible for color change in resin composite restorations. Researchers have conducted a few studies to evaluate the changes in surface roughness [8] and discoloration [8,9] of dental resin composite materials caused by vitamin syrups. Changes in the properties of resin composites, such as roughness and color, may affect the lifespan of these materials and increase patients’ esthetic concerns [10].

When assessing the color of teeth or restorative materials, there are many methods for measuring surfaces’ color, including visual comparisons using color scales, spectrophotometers, colorimeters, spectroradiometers, and digital image analysis techniques [11]. The spectrophotometer, which is one of the most frequently used devices for evaluating surface colors, measures the amount of light energy reflected from an object in the range of 1–25 nm on the visible spectrum. In addition to changes in color and measurement, it is also essential to acknowledge that the surface properties of resin composites and other glass-containing restorative materials change when exposed to strong acids. Increased surface roughness causes discoloration, plaque accumulation, gingival irritation, recurrent caries, susceptibility to abrasion, accelerated wear, and tactile roughness perception. An increase of 0.3 µm in surface roughness can be detected with the tongue. This sense of roughness leads to a decrease in patient comfort. In our study, the change on the resin composites’ surface caused by the organic acids in effervescent tablets was measured with a contact profilometer device [12,13]. Although the literature has extensively investigated the effects of various vitamins on dental hard tissues and acidic solutions on dental materials, there is a lack of knowledge regarding the effects of effervescent multivitamin tablets on discoloration and surface roughness of dental resin composite materials. Moreover, micro-hybrid resin composites are also among the most preferred materials for direct resin composite restorations. This in vitro study was intended to evaluate the effect of an effervescent tablet (Redoxon Triple Action, Bayer) with an acidic pH of 3.0 and containing vitamin C, vitamin D, and Zinc, taken daily for 30 days, on micro-hybrid resin composites’ surface roughness and color. The null hypothesis of the study was that different micro-hybrid resin composites do not differ in discoloration and surface roughness after 30 days of multivitamin exposure.

## 2. Materials and Methods

### 2.1. Preparation of Samples

A total of 50 disc-shaped (8 mm diameter × 2 mm thickness) light-cured micro-hybrid resin composite samples with shade A2 were prepared using a silicone mold, a Mylar transparent strip (Kerr Hawe, Bioggio, Switzerland) and a Teflon matrix using 5 different micro-hybrid resin composites (Dentac Pergamon/PM, Tokuyama Estelite Posterior/EP, GC Geanial Anterior/GA, Kulzer Charisma Opal/CO, Shofu Beautifil II/BII) listed in Table 1 (*n* = 10). A silicone mold was positioned over a glass side and a mylar strip and filled with composite in a single increment. The samples were polymerized for 20 s with a Valo Cordless (Ultradent, South Jordan, UT, USA) light-curing unit in the standard mode with a power output of 1100 mW/cm^2^, following the manufacturers’ guidelines. Following 4-step finishing and polishing procedures (Finishing Discs, Bisco, Schaumburg, IL, USA), samples were stored in deionized water at 37 °C (±1 °C) for 24 h as specified by the ISO/TR 28642: 2016 [14]. Initial color and surface roughness values were measured and recorded.

### 2.2. Immersion Procedure

**Figure 1:**
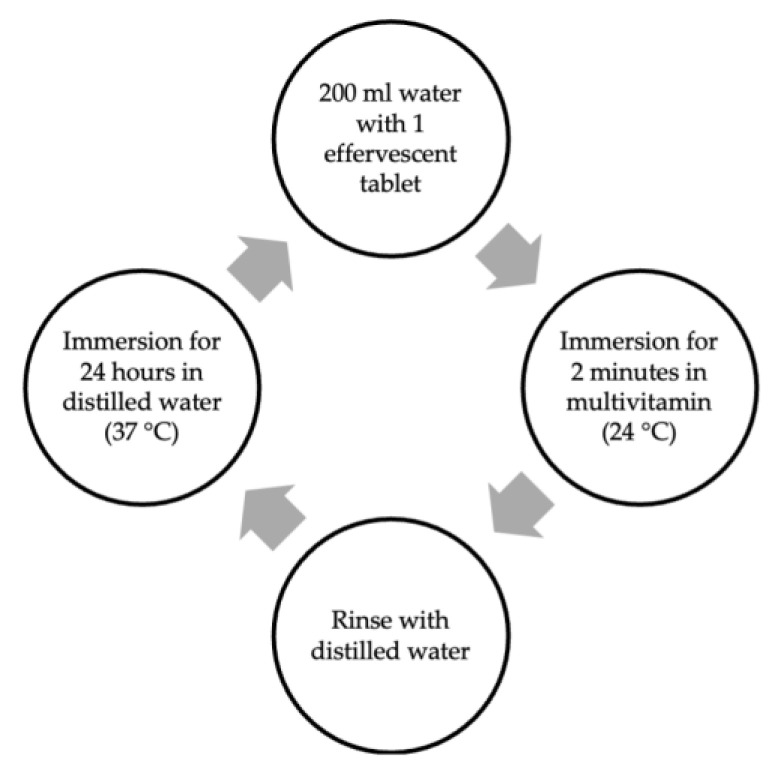
Immersion cycle in multivitamin solution for 30 days.

The samples of each micro-hybrid resin composite were randomly divided into two subgroups based on the immersion medium (Redoxon Triple Action effervescent and distilled water). Over 30 days, the control samples were kept in distilled water that was refreshed daily. The experimental samples were immersed in 200 mL of water (24 °C) with 1 effervescent tablet for 2 min a day in 24 h intervals between the immersion cycles (Figure 1).

Until the next cycle, they were stored in distilled water at 37 °C. The pH of the effervescent multivitamin was 3 in each immersion (Table 2).

The control group was immersed in distilled water (37 °C), which was refreshed at intervals of 24 h.

### 2.3. Color Measurement

The color was measured with a spectrophotometer (Easyshade V, VITA Zahnfabrik, Bad Säckingen, Germany) and on a gray background under the D65 standard illuminant. All color measurements were based on CIE L*a*b (International Commission on Illumination), and ΔEab values were calculated. The color difference (ΔE) or change was represented in the CIELab system and determined using the following formula [15]:

ΔE (L*a*b*) = ([ΔL*]^2^ + [Δa*]^2^ + [Δb*]^2^])^1/2^

where ΔL* is the difference between the L* values, Δa* is the difference between the a* values, and Δb* is the difference between the b* values.

The obtained ΔEab values were classified according to a review on acceptability and perceptibility thresholds [16].

### 2.4. Surface Roughness (Ra)

Surface roughness values were recorded at the beginning and at the end of the 30 days with a contact profilometer (SJ-201P, Mitutoyo, Kanagawa, Japan). The surface roughness readout was made over 0.5 mm with a cutoff value of 0.8 mm at a speed of 0.25 mm/s, and the resolution was 0.01 μm. Three different measurements in the same directions were recorded from the middle region of the top surface of each sample, and the mean of the measurements was calculated. The mean of the values was considered as the mean surface roughness (Ra) of the samples. The profilometer was calibrated before each new measurement session.

### 2.5. Scanning Electron Microscopy (SEM)

The surfaces of the composite samples were analyzed using an SEM (EVO-MA 10, Zeiss, Oberkochen, Germany) with an acceleration voltage of 10 kV under 1k and 5k magnifications. Prior to scanning and analyzing, the samples were coated with a thin layer of gold using a Quorum SC7620 sputter coater (Quorum Technologies, Laughton, UK) at 20 mA and 180 s.

### 2.6. Statistical Analysis

Data were analyzed with IBM SPSS V23. The conformity to the normal distribution was evaluated using the Shapiro–Wilk test. Analysis results are presented as mean ± standard deviation. The significance level was set as *p* < 0.050.

A two-way ANOVA was conducted to compare the ΔE and ΔL values, based on the composite type and vitamin solution, and multiple comparisons of the main effects were made with the Duncan test. A one-way ANOVA was conducted for multiple comparisons of significant composite interactions, and multiple comparisons were made with the Duncan and Tamhane tests.

The generalized linear model method was used to examine the effect of composite type, solution, and time main effects and interactions on surface roughness values, and multiple comparisons were made with the Bonferroni test.

## 3. Results

### 3.1. Discoloration

The main effect of the resin composite type was statistically significant for ΔE values (*p* = 0.040). The mean ΔE values differ depending on the brands of the resin composites (Table 3).

This difference arose due to the higher mean value for the BII resin composite. The solution’s main effect and resin composite/solution interaction were not statistically significant (*p* = 0.243, 0.281, respectively) (Table 4).

After being kept in distilled water, the CO group was classified as an “excellent match”. GA and EP were “acceptable matches”, PM was a “mismatch type a”, and BII was a “mismatch type b” based on 50:50% perceptibility and 50:50% acceptability thresholds. CO, GA, and PM were “acceptable matches” in groups immersed in a multivitamin for 30 days; EP was designated as a “mismatch type a”, and BII was a “mismatch type b” [16].

The mean ΔL values were 0.08 and −1.51 for distilled water and the vitamin solution, respectively. The highest mean ΔL value obtained was 1.43 for BII in distilled water, and the lowest was −4.35 for BII in the vitamin solution (Table 5).

Although the main effect of the micro-hybrid resin composite type did not show a significant effect on ΔL values (*p* = 0.419), it was determined that the resin composite type/solution interaction had a significant effect (*p* = 0.009). The main effect of the vitamin solution was statistically significant on ΔL values (*p* = 0.007) (Table 6).

### 3.2. Surface Roughness

The mean surface roughness values of PM and BII were significantly higher than those of EP, GA, and CO. While the total mean roughness value was 0.258 initially, it was measured as 0.317 on the 30th day (Table 7).

The resin composite type had a statistically significant effect on surface roughness values (*p* < 0.001). The main effect of the 30-day vitamin solution on the roughness values was statistically significant (*p* = 0.001) (Table 8).

### 3.3. SEM Evaluation

**Figure 2:**
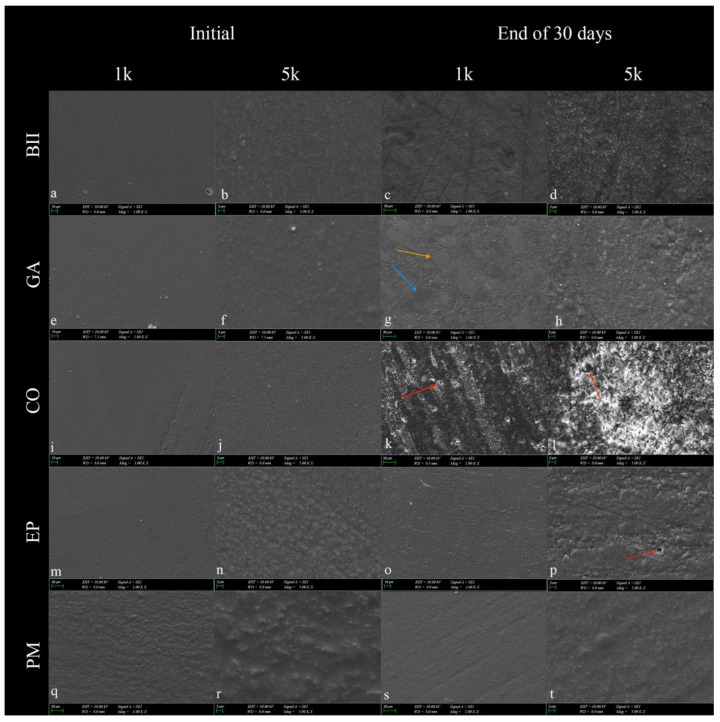
SEM images of micro-hybrid resin composites at 1000 (1k) and 5000 (5k) magnifications were taken before and after exposure to the multivitamin for 30 days. (**a**–**d**): Beautifil II; (**e**–**h**): Geanial Anterior; (**i**–**l**): Charisma Opal; (**m**–**p**): Estelite Posterior; (**q–t**): Pergamon. In resin composites, nanoclusters (orange arrow) and prepolymerized fillers (blue arrow) became prominent following exposure to the vitamin solution. Signs of fallout of the fillers and decomposition of the matrix were observed following immersion in the vitamin solution (red arrow). Clusters of debris formed from loose mineral particles were observed in (**d**,**h**,**l**,**p**,**t**). The filler sizes in the images were consistent with the manufacturer’s information on the content (Table 1).

Exposure to an acidic vitamin solution resulted in a rougher and heterogeneous surface in all micro-hybrid resin composites after 30 days. Especially in ×5.000 images, small cracks and pits on the resin composite surfaces were detected. Prepolymerized fillers and nanoclusters were visible on the surface of GA after acidic exposure. Signs of fallout of the fillers were observed in CO and EP. In the initial stage, the PM resin composite appeared rougher than the others (Figure 2).

## 4. Discussion

Resin composites with various filler contents and sizes are used in restorative dentistry. One of the most used types is micro-hybrid resin composites, which are known to provide optimum mechanical, optical, and physical properties. The amount of smoothness/roughness in these resin composite materials affects optical properties, discoloration, water sorption, plaque accumulation, and periodontal tissue health [17]. The discoloration of resin composite restorations may cause loss of esthetic properties and additional costs for the renewal of the restoration. At the same time, a rough surface causes wear and shortens the material’s life [10,18]. Discoloration in resin composites, in addition to extrinsic factors, is also caused by resin-matrix filler particles, particle-matrix boundaries, and resin monomer absorption. The filler particle size and filler concept also affect resin composites’ surface roughness [19,20]. Additionally, studies have indicated that acidic solutions can affect the roughness of resin composites [6,21]. Moreover, water absorption occurs because of the space between molecules in the resin matrix [22,23]. Glass-filler particles are generally inert, so it is thought that they do not tend to absorb fluids. Resin percentage and hydrophilic co-monomers in the structure also affect color instability [24]. High filler and low monomer content promotes less water absorption because monomers demonstrate high hydrophilicity. Unreacted monomers cause more liquid absorption, and the degree of conversion of some monomers under the same conditions is as follows: Bis-GMA < Bis-EMA < UDMA < TEGDMA [25,26,27]. This study used Bis-GMA in all resin composites except for Geanial Anterior. The Charisma Opal composite does not contain TEGDMA. Beautifil II stands out from the others as it contains S-PRG. The polarimerization difference between resin polymers and pigments can also cause the absorption of yellow pigments by the organic part in resin materials [28]. Redoxon Triple Action multivitamin also contains beta-carotene-derived yellow colorant according to the manufacturer. However, no studies have been conducted on its staining properties and this study planned to investigate the effect of an effervescent multivitamin on color and surface roughness.

For color evaluation, a Vita Easyshade V was used in this study because it provides objective, repeatable, and fast measurements [29]. For this reason, in this study using a spectrophotometer, the following subgroups were seen in the classification made with the calculated ΔEab. Although there was no difference, only Charisma Opal was “excellent acceptable” when kept in distilled water according to the acceptability/perceptibility threshold table. This result can be attributed to the fact that Charisma Opal contains only Bis-GMA, whereas other micro-hybrid resin composites contain UDMA and TEGDMA and undergo discoloration due to water absorption. Among the samples immersed in a multivitamin containing vitamin C, the Beautifil II was mismatch type b (clearly unacceptable) [16], possibly due to the S-PRG in it. Giomers contain Bis-GMA and TEGDMA, similar to resin composites, and they are prone to fluid absorption. In an in vitro study, the color stability of the S-PRG of the giomer was investigated. When immersed in a solution, the S-PRG made the surface more water-absorbent, thereby balancing internal and external pressure [30,31].

Almutairi et al. also included multivitamins in their study, in which they used popular pediatric fluid medications. They found that GIC (glass ionomer cement) was less color stable [9]. Similarly, the giomer was the most discolored material in the present study due to its glass ionomer content. Gönülol et al. found that giomer material exhibited the most significant color changes in comparison to nano-hybrid resin composites in their study [31]. Adusumilli et al. reported that the color change was higher in GIC than in the giomer in their study with various foods and beverages and various durations [32]. The giomer used in the present study showed more discoloration than the micro-hybrid resin composites, possibly due to the glass ionomer structure. The giomer Beautifil II containing S-PRG used in this study may have shown more color change with the yellow-pigmented vitamin solution because it is more susceptible to liquid absorption. With water absorption, teeth and restorations were dipped in various aqueous solutions in the mouth and underwent continuous erosion attacks. An external or biofilm-induced decrease in pH can be seen in the oral environment, resulting in the deterioration of surface integrity and, consequently, increased sensitivity to staining [33,34,35]. As previously noted by researchers, surface roughness and coloration are interrelated terms. In this study, the higher surface roughness of giomers prior to immersion in the vitamin solution than that of the other resin composites may have caused more color change. In the present study, the solutions’ pH was measured as threefor 30 days. Both the surface roughness and the color of the micro-hybrid resin composites were affected by the acidic solution containing yellow colorants. Still, the null hypothesis of the study was partially accepted since no significant difference in surface roughness or discoloration was observed between materials (except Beautifil II). However, the result may have been influenced by the initial surface roughness of Beautifil II. In a surface roughness assessment, Chung reported that the surface roughness value, determined by 2D profilometry, was less than 1 μm when the resin composite surfaces were visibly smooth [36]. However, if the 2D surface roughness exceeds 0.2 μm, it surpasses the clinically acceptable threshold for resin composite restorations [37]. In the present study, after 30 days of immersion in multivitamins, neither composite met the acceptable threshold. The resin composites immersed in the multivitamin showed differences in surface roughness in comparison to the control group. However, the control group showed changes in surface roughness after 30 days, and some resin composite samples did not possess acceptable surface roughness even at the initial stage. Additionally, there were also different surface roughness values of resin composites in the same group that varied initially. This might be due to the selected area (center of the specimen) or the possibility of voids in the material [38], even if standard procedures were followed during the preparation of samples.

The filler volume of the resin composite is linearly related to the surface’s abrasion resistance to acid attacks [39]. Han et al. found that flowable composites with a higher filler content have greater resistance to acidic substances [40]. Similarly, SEM evaluations in this study revealed the least matrix degradation and filler fallout on the Pergamon surface due to its higher filler ratio. These were observed more clearly in the Charisma Opal composite with the lowest filler content by volume (Figure 2). Resins serve to protect against hydrolytic degradation to some extent as a coupling agent in resin composites. Therefore, an organic matrix structure that is not sufficiently polymerized can dissolve in any acidic solution, and filler fallout may observed [41]. The filler particles on a composite surface that are exposed tend to erode at pH = 3, resulting in their loss and the formation of surface dimples, which may increase surface roughness. A previous study mentioned the effect of filler mineral loss because of acidic erosion of the surface [42]. Considering the increase in surface roughness as a result of this study, loose mineral particles form debris clusters that might be combined with pigments from the multivitamin solution, forming stain scales (Figure 2) which also affect the surface roughness value.

Different from the present study, Kaya et al. showed SEM images of large and rectangular glass particles embedded in a giomer resin structure [21]. However, according to this study, all resin composite surfaces appeared rougher in SEM images at the end of the 30-day multivitamin cycle, compared to their initial images, parallel to the surface roughness values. Iosif et. al. stated that exposure to an acidic environment reveals good preservation of orthodontic cement samples’ microstructure, however the outermost unevenness is eroded [42]. The researchers’ results show similarities to the effects of acidity on both the surface roughness and SEM images of the micro-hybrid resin composites in the present study.

In surface topography assessments, errors such as noise and flat deviation are influential factors that should be considered when evaluating the results of this study [43]. The surface properties of resin composites are related to their degree of conversion, but this aspect was not evaluated in this study. An acidic solution was preferred in this study, but the degree of conversion should also be evaluated to get an idea of the resin composites’ behavior in reaction to acidic solutions. The CIELab formula was used for color evaluation in this study. CIEDE2000 and CIELab formulas are commonly used in the stainability of restorative materials [44,45,46]. Although CIEDE2000 is known to be more closely correlated with human visual observations [47], Lee investigated two formulas and found that they were correlated [48]. Additionally, the effect of longer use of vitamins on resin composite restorations could be investigated.

### The Outlook

Future studies are required to evaluate the mechanical properties, surface wear, and sorption and solubility of resin composite materials under challenging conditions in the oral cavity.

## 5. Conclusions

This study attempted to reflect the intake of effervescent multivitamin tablets once daily for 30 days. Under the limitations of this in vitro study, the conclusions are as follows:

- The effervescent multivitamin tablet had an impact on the color of the giomer. However, the multivitamin solution did not affect the color of other micro-hybrid resin composites.
- The multivitamin tablet with a pH of 3.0 is thought to have various effects on the surface roughness, depending on the content of the tested micro-hybrid resin composites. The largest change in surface roughness was detected in the giomer, similar to discoloration.
- Material selection is important for patients who regularly consume multivitamin tablets with an acidic pH.

## Figures and Tables

**Table 1:** The composition of the micro-hybrid resin composites used in the study.

Product Name	Manufacturer	Resin Composition	Filler (Volume%)	Shade/ Lot Number
Beautifil II (BII)	Shofu Dental, Menlo Park, CA, USA	Bis-GMA, TEGDMA, S-PRG filler based on fluoroboroaluminosilicate glass (0.01–4.0 µm), polymerization initiator, pigments, and others.	68.6%	A2/031917
Geanial Anterior (GA)	GC, Tokyo, Japan	UDMA, dimethacrylate co-monomers, prepolymerized fillers containing silica, prepolymerized particles containing strontium and lanthanoid fluoride, silica, fumed silica (0.1–17 µm).	64%	A2/190204A
Pergamon (PM)	Dentac, Istanbul, Turkey	Bis-GMA, TEGDMA, inorganic fillers silica quartz 0.5–0.7 µm, pigments and initiators.	77–78%	A2/20210204
Estelite Posterior (EP)	Tokuyama, Tokyo, Japan	Silica-zirconia filler (0.1–10 µm (2 µm)), Bis-GMA, TEGDMA, Bis-MEPP, Radical-Amplified Photopolymerization initiator technology.	70%	A2/W135
Charisma Opal (CO)	Kulzer, Hanau, Germany	Bis-GMA matrix, barium aluminum glass (0.02–2 µm), highly dispersive silica (0.02–0.07 µm).	58%	A2/KA10705

Abbreviations, Bis-GMA: Bisphenol A glycidyl methacrylate, TEGDMA: Triethylene glycol dimethacrylate, S-PRG: Surface-prereacted glass-ionomer, Bis-MEPP: Bisphenol A polyethoxy methacrylate.

**Table 2:** Contents of the effervescent multivitamin.

Redoxon Triple Action (Bayer)	Contents
Vitamin C (Ascorbic acıd)	1000 mg (1 tablet)
Vitamin D (Cholecalciferol)	400 IU (10 mcg) (1 tablet)
Zinc (Zinc citrate)	10 mg (1 tablet)
Other	Acids (Citric acid, Malic acid), Acidity regulators (Sodium carbonates), Bulking agent (Isomalt), Color (Beta-carotene), Flavoring, Sweeteners (Acesulfame K, Sucralose), Salt.

**Table 3:** Descriptive statistics of ΔE_ab_ values by resin composite type and vitamin solution.

	CO	GA	EP	BII	PM	Main Effect *
Distilled Water	0.48 (±0.40)	1.27 (±0.61)	1.49 (±0.21)	3.49 (±1.61)	2.08 (±1.95)	1.76 (±1.49)
Vitamin Solution	1.77 (±1.21)	1.58 (±0.58)	1.89 (±1.15)	3.98 (±0.49)	1.29 (±0.35)	2.10 (±1.24)
Main effect **	1.13 (±1.09 ^b^)	1.42 (±0.58 ^b^)	1.69 (±0.81 ^b^)	3.73 (±1.15 ^a^)	1.68 (±1.38 ^b^)	1.93 (±1.37)

Mean (±standard deviation), a–b: the same letter is not significantly different, * solution main effect regardless of the resin composite type (*p* = 0.243), **: resin composite type main effect regardless of the solution (*p* = 0.000).

**Table 4:** Comparison of ΔE_ab_ values according to resin composite type and vitamin solution.

Group		Sum of Squares	Sd	Mean of Squares	F	*p*	Partial Eta Square
Resin Composites	Resin Composite type	42.738	4	10.685	10.236	0.000	0.506
	Solution	1.467	1	1.467	1.405	0.243	0.034
	Resin Composite Type × Solution	5.489	4	1.372	1.315	0.281	0.116

R^2^ = 0.543, corrected R^2^ = 0.441, Sd: degrees of freedom, F: analysis of variance test statistics.

**Table 5:** Descriptive statistics of ΔL values by resin composite type and vitamin solution.

	CO	GA	EP	BII	PM	Main Effect *
Distilled Water	0.05 (±0.46 ^B^)	−0.42 (±1.12 ^B^)	−0.49 (±1.36 ^AB^)	1.43 (±1.17 ^B^)	−0.14 (±3.62 ^AB^)	0.08 (±1.86)
Vitamin solution	0.35 (±0.61 ^B^)	−1.50 (±1.71 ^AB^)	−0.38 (±1.23 ^B^)	−4.35 (±0.46 ^A^)	−1.67 (±4.05 ^AB^)	−1.51 (±2.50)
Main effect **	0.20 (±0.53)	−0.96 (±1.48)	−0.44 (±1.22)	−1.46 (±3.16)	−0.91 (±3.71)	−0.71 (±2.32)

Mean (±standard deviation), A–B: the same letter is not significantly different, *: solution main effect regardless of the resin composite type (*p* = 0.007), **: resin composite type main effect regardless of the solution (*p* = 0.419).

**Table 6:** Comparison of ΔL values according to resin composite type and vitamin solution.

Group		Sum of Squares	Sd	Mean of Squares	F	*p*	Partial Eta Square
Resin Composites	Resin Composite Type	15.661	4	3.915	1.000	0.419	0.091
	Solution	31.729	1	31.729	8.103	0.007	0.168
	Resin Composite Type × Solution	60.641	4	15.16	3.872	0.009	0.279

R^2^ = 0.562, corrected R^2^ = 0.480; Sd: degrees of freedom, F: analysis of variance test statistics.

**Table 7:** Descriptive statistics of surface roughness (μm) values of resin composites.

Solution	Time	Resin Composite					Main Effect *
		EP	GA	PM	CO	BII	
Distilled Water	Initial	0.19 (±0.02)	0.16 (±0.04)	0.38 (±0.15)	0.20 (±0.06)	0.33 (±0.05)	0.25 (±0.11)
	Day 30	0.20 (±0.03)	0.22 (±0.05)	0.41 (±0.16)	0.26 (±0.12)	0.44 (±0.13)	0.30 (±0.14)
	Total	0.19 (±0.02)	0.19 (±0.05)	0.39 (±0.15)	0.23 (±0.09)	0.38 (±0.10)	0.28 (±0.13)
Vitamin Solution	Initial	0.26 (±0.05)	0.26 (±0.06)	0.26 (±0.08)	0.19 (±0.05)	0.33 (±0.09)	0.26 (±0.08)
	Day 30	0.23 (±0.11)	0.27 (±0.10)	0.40 (±0.11)	0.23 (±0.14)	0.52 (±0.15)	0.33 (±0.16)
	Total	0.25 (±0.08)	0.27 (±0.08)	0.33 (±0.11)	0.21 (±0.01)	0.42 (±0.16)	0.30 (±0.13)
Main effect **	Initial	0.23 (±0.06)	0.21 (±0.07)	0.32 (±0.13)	0.20 (±0.05)	0.33 (±0.07)	0.26 (±0.10)
	Day 30	0.22 (±0.08)	0.25 (±0.08)	0.40 (±0.13)	0.25 (±0.12)	0.48 (±0.14)	0.32 (±0.15)
	Total	0.22 (±0.07 ^b^)	0.23 (±0.07 ^b^)	0.36 (±0.13 ^a^)	0.22 (±0.09 ^b^)	0.40 (±0.13 ^a^)	0.29 (±0.13)

Mean (±standard deviation), a–b: the same letter is not significantly different, *: solution main effect regardless of resin composite type, **: resin composite type main effect regardless of the solution.

**Table 8:** Comparison of roughness values according to resin composite.

		Test Statistics *	Sd	*p*
Resin Composites	Resin Composite Type	83.542	4	**<0.001**
	Vitamin Solution	1.067	1	0.302
	Time (30 days)	11.946	1	**0.001**
	Resin Composite Type × Vitamin Solution	8.733	4	0.068
	Resin Composite Type × Time	9.084	4	0.059
	Vitamin Solution × Time	0.306	1	0.580
	Resin Composite Type × Vitamin Solution × Time	3.357	4	0.500

* Wald Chi-square test statistic, Sd: degrees of freedom.

## Data Availability

The data contained within the article are available on request from the corresponding author.
